# Unintended pregnancy with retained intrauterine balloon after hysteroscopic adhesiolysis: a case report

**DOI:** 10.3389/fmed.2025.1685692

**Published:** 2025-12-04

**Authors:** Yan Liao, Changqiang Xue, Yibao Huang, Yongxin Sun, Qingmei Feng, Yeping Wei, Liying Zhang

**Affiliations:** 1 Department of Gynecology, The Second Affiliated Hospital of Guangxi Medical University, Nanning, China; 2 Department of Human Anatomy, School of Basic Medical Sciences, Guangxi Medical University, Nanning, China; 3 Key Laboratory of Human Development and Disease Research, Guangxi Medical University, Education Department of Guangxi Zhuang Autonomous Region, Nanning, China

**Keywords:** intrauterine adhesions, hysteroscopic adhesiolysis, balloon placement, unintended pregnancy, case report

## Abstract

**Objective:**

To report a rare case achieving successful pregnancy with retained intrauterine balloon.

**Design:**

Case report.

**Setting:**

Obstetrics and Gynecology, The Second Affiliated Hospital of Guangxi Medical University, China.

**Patient:**

A 24-year-old female diagnosed with intrauterine adhesions unexpectedly conceived with retained intrauterine balloon.

**Main outcome measures:**

Successful pregnancy during balloon placement.

**Results:**

After regular prenatal check, the patient finally delivered a healthy baby through caesarean section without adverse pregnancy outcomes.

**Conclusion:**

Hysteroscopic adhesiolysis combined with Cook balloon placement is an effective approach for treating intrauterine adhesions. During balloon placement, strict contraception is required.

## Introduction

Intrauterine adhesions constitute a prevalent gynaecological disorder resulting from endometrial basal layer damage caused by uterine trauma. This damage can lead to partial or complete occlusion of the uterine cavity. The clinical manifestations vary among patients according to the location, severity, and extent of intrauterine adhesions, which commonly present as oligomenorrhea, amenorrhea, and recurrent miscarriages or infertility in severe cases (1).

Hysteroscopic adhesiolysis has proven effective in treating moderate to severe intrauterine adhesions. However, patients are prone to a relatively high recurrence rate (2). The Cook balloon, made of silicone to mimic the uterine cavity, can effectively isolate the uterine wall from the wound area. It facilitates drainage, applies compressive hemostasis, and minimizes infection risk (3). The soft surface of the Cook balloon protects the damaged endometrium, allowing the repair and regeneration of endometrium on its surface, thus providing notable efficacy in adhesion prevention (4). Therefore, hysteroscopic adhesiolysis with Cook balloon placement is an effective approach for promoting menstrual recovery, preventing readhesion, and improving pregnancy outcomes.

Pregnancy is unlikely to occur during the placement of intrauterine balloons. The balloon occupies the uterine cavity, potentially hindering sperm transport and fertilized egg implantation, and reducing the likelihood of natural conception (5). Therefore, the need for strict contraception is frequently overlooked after adhesion surgery. This case report describes the clinical course of a successful pregnancy with a retained intrauterine balloon. To date, there are no documented reports of pregnancy during the intrauterine balloon retention period, and its safety and pregnancy outcomes remain uncertain.

## Case presentation

**Figure 1:**
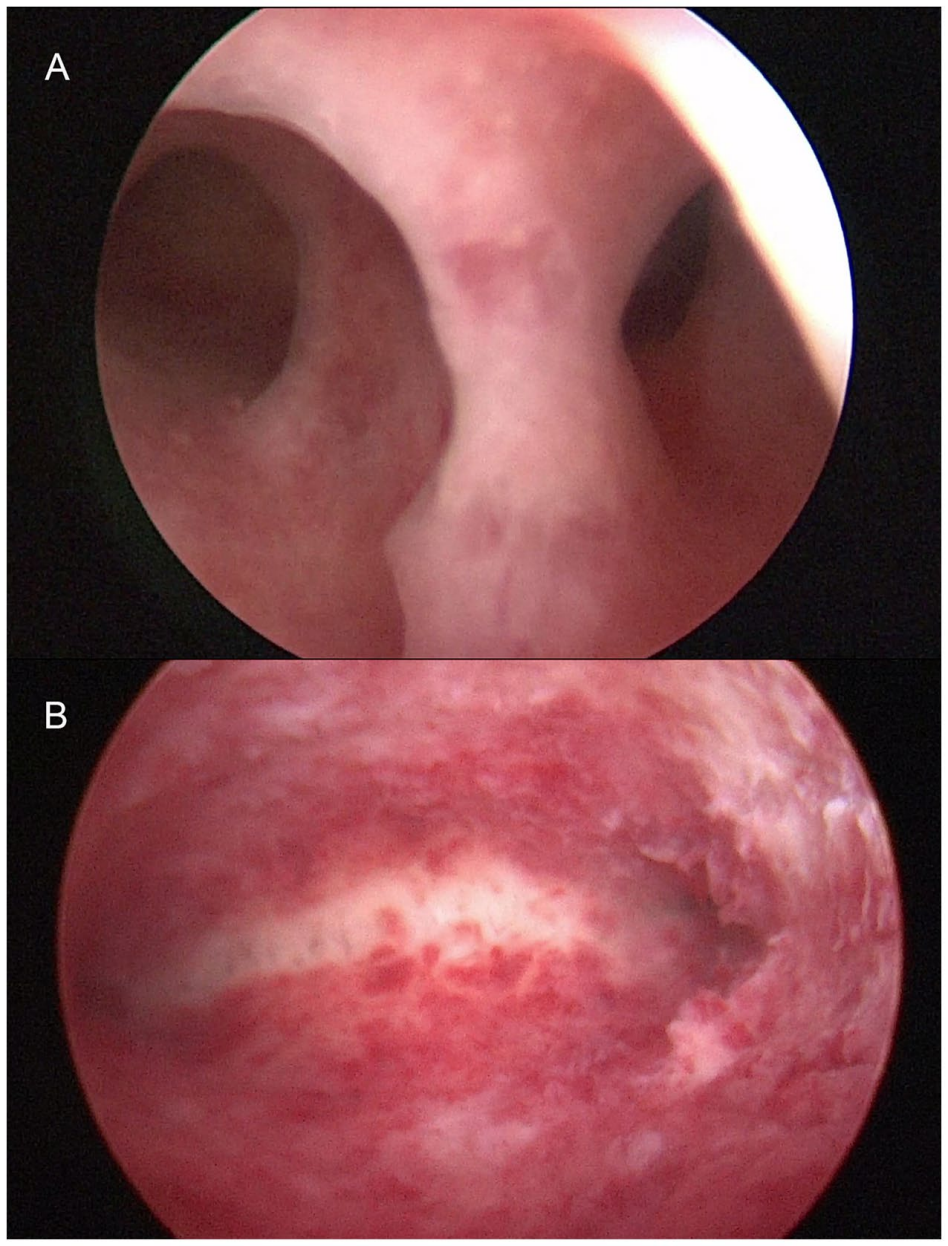
Moderate intrauterine adhesion under hysteroscopy, with preoperative **(A)** and postoperative **(B)** picture.

**Figure 2:**
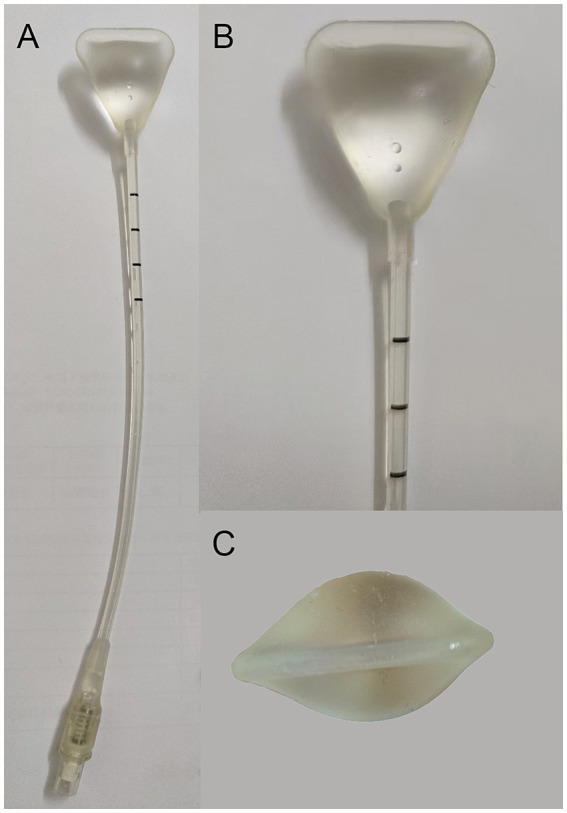
The Cook balloon is a triangular device with a single catheter **(A)**, which was inflated to a volume of 3 mL using saline **(B,C)**.

A 24-year-old female, G3P0A3, was admitted to our hospital with a 6-month history of oligomenorrhea. A gynaecological ultrasound revealed continuous interruption of the endometrium with an endometrial thickness of 0.7 cm. After informed consent was obtained, hysteroscopic adhesiolysis was performed, followed by Cook balloon placement. A 0.3 cm membranous adhesion was present at the internal cervical os and another in the mid-to-posterior cavity. The endometrium was thin and pink and the cavity contour irregular. The cornu and tubal ostium bilaterally were visible (Figure 1A). The cervical and intrauterine adhesions were dissected bluntly and sharply with cold scissors, after which the cavity returned to its normal inverted triangular shape (Figure 1B). To prevent adhesion recurrence, a Cook balloon, a triangular-shaped device with a single catheter, was placed in the uterine cavity and inflated with 3 mL of saline (Figure 2). Following the intraoperative assessment, the American Fertility Society (AFS) score was determined to be 5 points. Therefore, moderate intrauterine adhesion was diagnosed. The patient further received sequential oestrogen and progestogen therapy.

**Figure 3:**
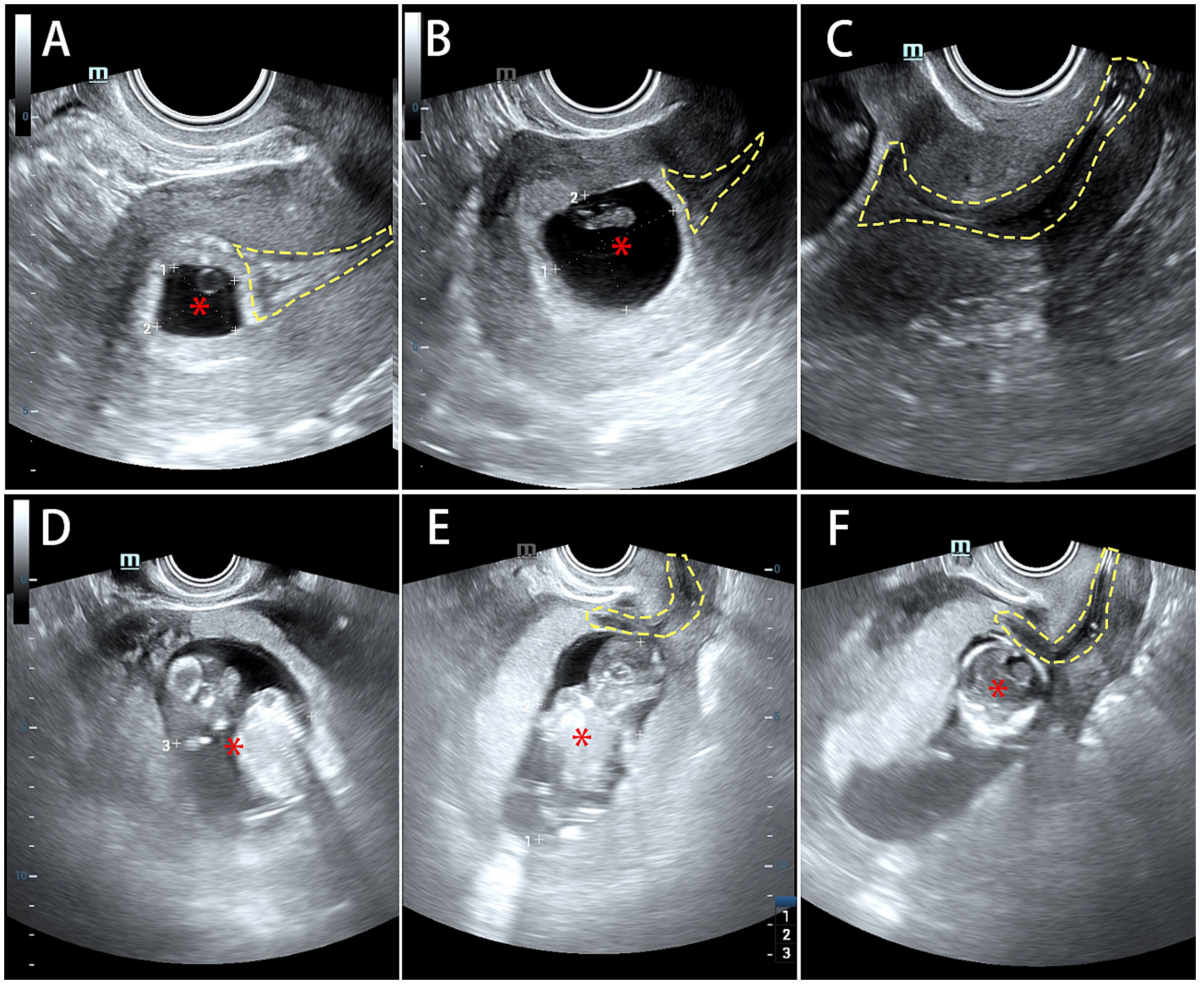
The follow-up ultrasound showed the foetal and its appendages developed normally. **(A)** A 1.5 × 1.5 × 1.9 cm gestational sac echo in the uterus that contained a visible embryo measuring 0.2 cm in length with detectable heartbeats. **(B)** An echo of gestational sac (asterisk) was observed in the uterus, measuring 2.8 × 2.4 × 2.6 cm in size, with a 1.4 cm embryo, corresponding to approximately a 7-week pregnancy. **(C)** The echo of Cook balloon (dashed line) was normal in position, but close proximity to the developing gestational sac. **(D,E)** An echo of gestational sac was seen in the uterus, measuring 7.5 × 4.7 × 3.6 cm in size, with an embryo outline inside (asterisk), equivalent to approximately 13 weeks of pregnancy. The placenta implants on the anterior wall. **(F)** The echo of Cook balloon (dashed line) could be seen inside the cervical canal.

After 50 days, the patient’s menstruation normalised, with an eight-day menstrual duration and moderate volume. The endometrial thickness was 0.8 cm on ultrasound. It was recommended that the balloon be removed two days after the next menstrual period ended. However, the patient reported engaging in sexual activity without contraception and then experienced amenorrhea for 2 months. Therefore, she sought medical attention and tested positive for serum β-HCG (29601.74 mIU/mL). In addition, gynaecological ultrasound revealed a 1.5 × 1.5 × 1.9 cm gestational sac echo in the uterus that contained a visible embryo measuring 0.2 cm in length with detectable heartbeats (Figure 3A). The patient was adamant about preserving the pregnancy and chose to continue the pregnancy. After providing comprehensive details regarding the risks associated with both balloon removal and retention, the patient underwent regular prenatal checks. Follow-up ultrasound revealed that the foetus along with its appendages developed normally, without any adverse pregnancy outcomes, such as placenta previa, chorioamnionitis, or miscarriage. In addition, the echo of the Cook balloon revealed a normal position, but close proximity to the developing gestational sac (Figures 3B–F). Ultimately, the patient delivered a healthy baby at full term via caesarean section, at which time the complete balloon was removed. Appropriate written consent for publication of the case was obtained.

## Discussion

This is the first case of a successful pregnancy with a retained intrauterine balloon after hysteroscopic adhesiolysis. In the current patient, the Cook balloon showed notable safety and efficacy in readhesion prevention and endometrium repair. Therefore, hysteroscopic adhesiolysis combined with Cook balloon placement could effectively treat moderate to severe intrauterine adhesions.

Intrauterine adhesions, a common gynaecological condition caused mainly by repeated abortions or curettage, are a major cause of infertility (2). Abortion surgery or curettage can cause mechanical damage to the endometrial basal layer, and the risk of intrauterine adhesions is proportional to the number of such surgeries (6). In addition, the live birth rate decreases following one, two or more miscarriages from initial rates of 74 to 67 and 58%, respectively (7). This patient had undergone three previous abortion surgery, with an AFS score of 5, and experienced oligomenorrhea and a heightened risk of infertility.

To prevent the development of intrauterine adhesions, researchers have devised and refined several interventions. First, an intrauterine contraceptive device can be placed for adhesion prevention (8). In addition, a Foley catheter can be inserted into the uterine cavity following hysteroscopic surgery to prevent the recurrence of intrauterine adhesions. Similarly, a triangular intrauterine balloon, such as Cook balloon, which perfectly adapts to the uterine cavity’s structure and maintains separation along its margins, is also frequently used for adhesion prevention (9). Currently, sequential oestrogen and progestogen therapy is commonly used to promote endometrial proliferation and healing after hysteroscopic surgery (10). In this patient, a comprehensive approach incorporating all of these therapies was used. After the sequential oestrogen and progestogen therapy, she resumed menstruation, which was accompanied by satisfactory endometrial thickness.

However, the patient engaged in sexual activity without contraception, leading to an unintended pregnancy with a retained uterine balloon. To date, no documented cases of pregnancy with a retained uterine balloon have been reported. This case emphasizes the necessity of strict contraception during balloon placement. The current guidelines and product manuals should emphasize the importance of abstinence or barrier contraception during balloon catheterization until the first menstrual cycle resumes after balloon removal. In addition, preoperative conversations should inform the patient of the risk of unplanned pregnancy during balloon placement.

After careful multidisciplinary evaluation, it was determined that the balloon was positioned in close proximity to the developing gestational sac. Given the risk of mechanical disruption to the gestational sac or triggering uterine contractions during balloon extraction, the removal procedure was determined to have a high risk of pregnancy loss. The patient was fully counseled regarding these uncertainties and the potential risks associated with both removal and retention. After thorough discussion, she expressed a strong preference to avoid any invasive intervention that may jeopardize the pregnancy and opted for expectant management under close surveillance. Fortunately, she delivered a healthy baby at full term via caesarean section without adverse outcomes. This is the first case of pregnancy with a retained intrauterine balloon, a scenario that is more uncommon than pregnancy with an intrauterine device (IUD). A previous study utilizing data from nationwide inpatient sample revealed that the pregnancy rate with an IUD was 0.02% (11), supporting that intrauterine balloon *in situ* is a rare complication of pregnancy. Several studies have confirmed that pregnancy with IUD increases the risk of miscarriage, stillbirth, premature birth, premature membrane rupture, and chorioamnionitis (12, 13). In this case, the long-term health outcomes for the child remain unknown. Due to potential risks, maintaining the pregnancy during balloon placement is not advised.

## Conclusion

Hysteroscopic adhesiolysis combined with Cook balloon placement is an effective approach for treating intrauterine adhesions. During intrauterine balloon placement, sexual activity is prohibited, and strict contraception is required, especially once endometrial function normalizes. It is still not advisable to maintain pregnancy due to potential risks.

## Capsule

This is the first case of a successful pregnancy with retained intrauterine balloon after hysteroscopic adhesiolysis.

## Data Availability

The original contributions presented in the study are included in the article/supplementary material, further inquiries can be directed to the corresponding author.
